# CD4^+^CD25^+ ^T regulatory cells from FIV^+ ^cats induce a unique anergic profile in CD8^+ ^lymphocyte targets

**DOI:** 10.1186/1742-4690-7-97

**Published:** 2010-11-19

**Authors:** Jonathan E Fogle, Wayne A Tompkins, Mary B Tompkins

**Affiliations:** 1 North Carolina State University, College of Veterinary Medicine, Immunology Program, Department of Population Health and Pathobiology, 4700 Hillsborough Street, Raleigh, NC, USA 27606

## Abstract

**Background:**

Using the FIV model, we reported previously that CD4^+^CD25^+ ^T regulatory (Treg) cells from FIV^+ ^cats are constitutively activated and suppress CD4^+^CD25^- ^and CD8^+ ^T cell immune responses. In an effort to further explore Treg-mediated suppression, we asked whether Treg cells induce anergy through the alteration of production of cyclins, cyclin-dependent kinases and their inhibitors.

**Results:**

Lymphocytes were obtained from control or FIV^+ ^cats and sorted by FACS into CD4^+^CD25^+ ^and CD8^+ ^populations. Following co-culture with CD4^+^CD25^+ ^cells, CD8^+ ^targets were examined by Western blot for changes in cyclins D_3_, E and A, retinoblastoma (Rb) protein, as well as the cyclin dependent kinase inhibitor p21^cip1^. Following co-culture with CD4^+^CD25^+^cells, we observed up-regulation of p21^cip1 ^and cyclin E, with down-regulation of cyclin D_3_, in CD8^+ ^cells from FIV^+ ^cats. As expected, CD8^+ ^targets from control cats were quiescent with little up-regulation of p21^cip1 ^and cyclin E. There was also a lack of Rb phosphorylation in CD8^+ ^targets consistent with late G_1 _cell cycle arrest. Further, IL-2 mRNA was down regulated in CD8^+ ^cells after co-culture with CD4^+^CD25^+ ^Treg cells. Following CD4^+^CD25^+ ^co-culture, CD8^+ ^targets from FIV^+ ^cats also had increased Foxp3 mRNA expression; however, these CD8^+^Foxp3^+ ^cells did not exhibit suppressor function.

**Conclusions:**

Collectively, these data suggest that CD4^+^CD25^+ ^Treg cells from FIV^+ ^cats induce CD8^+ ^anergy by disruption of normal G_1 _to S cell cycle progression.

## Background

Using FIV as an AIDS lentivirus model, we reported previously that CD4^+^CD25^+ ^Treg cells in both the acute phase and long-term, asymptomatic phase of infection are constitutively activated and suppress CD4^+^CD25^- ^and CD8^+ ^T cell immune responses [1-3]. Activated feline Treg cells from FIV^+ ^cats suppress CD4^+ ^cell proliferation and IL-2 production and CD8^+ ^cell IFNγ production [1,3,4]. We have demonstrated preferential in vitro and in vivo replication of FIV in the CD4^+^CD25^+ ^subset, suggesting a unique relationship between lentiviral infections and Treg cell activation [4,5]. Impaired CD8^+ ^T cell immune responses are well described in AIDS lentivirus infections and evidence suggests that this impairment correlates with activation of CD4^+^CD25^+ ^Treg cells [6-9].

Lentivirus infections are characterized by an early increase in CD8^+ ^T lymphocyte numbers, and the quality of the CTL response is associated with a decline in plasma viremia. A strong CTL response correlates with clearance of virus from circulation, and a weaker response is associated with poor or no control of viral replication [10-15]. Experimental models and clinical data from other types of viral infections have clearly demonstrated that CD8^+ ^lymphocytes are critical for the control of viral infection, and escape of this initial response can lead to establishment and maintenance of a persistent infection and may contribute to immune exhaustion [16-22]. Using the FIV model we designed experiments to identify lentiviral mechanism(s) used to escape virus elimination and establish a chronic infection in the face of a robust CD8^+ ^response. These experiments have focused on Treg cell activation kinetics during FIV infection, the mechanism of Treg mediated suppression, and identification of cells targeted for Treg-mediated suppression; and we have clearly established that Treg cells are able to suppress CD8^+ ^effector responses during both acute and chronic FIV infection [1-3]. We therefore asked what intracellular events occur in the CD8^+ ^target cell following interaction with CD4^+^CD25^+ ^Treg cells, do these intracellular events contribute to CD8^+ ^anergy, and could these CD8^+ ^targets be converted into CD8^+ ^suppressor cells?

**Figure 1:**
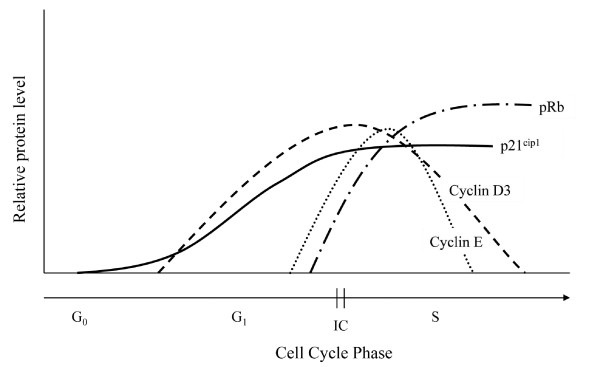
**A schematic representation of the relative protein levels during the normal progression from G_1 _to S phase of the cell cycle**. In T lymphocytes during the normal progression of the cell cycle, D cyclins (D_2 _and D_3_, D_2 _not shown) are expressed sequentially starting in late G_0_/early G_1_. At approximately the same time the relative level of the CDKI p21^cip1 ^begins to increase and then plateaus during late G_1_/early S phase. p21^cip1 ^inhibits cyclin E until the cellular machinery is ready for a synchronized G_1 _to S phase transition. Cyclin E levels begin to rise during late G_1_and peak during early S phase. Separation of Rb from E2F proteins and hyperphosphorylation of Rb at multiple sites signals the irreversible commitment (IC, double line) to S phase and cell cycle progression. (Note: only the proteins examined in Figures 2-5 are represented here.)

Down-regulation of IL-2 production, loss of effector function, and lack of proliferation are well described in lymphocyte target cells following interaction with activated CD4^+^CD25^+ ^Treg cells [1,23-25]. However, these events are the end result of a complex process, including interruption of cell cycling events, that may occur in CD4^+^CD25^- ^or CD8^+ ^target cells following their interaction with CD4^+^CD25^+ ^Treg cells. Cell cycle progression is tightly regulated by proteins such as cyclins, cyclin dependent kinases (CDKs) and cyclin dependent kinase inhibitors (CDKIs) that ensure an appropriate and coordinated cellular response. This mechanism responds to intracellular and extracellular signals and will arrest cell cycle progression (induce anergy) in response to adverse intracellular or extracellular conditions [26]. During the early immune response, primary T lymphocytes that receive optimal stimulation through their TCR and co-stimulatory pathways proceed through G_1 _cell cycle progression (Figure 1). Subsequent multiple cell divisions are then required during this primary response for optimal IL-2 and IFNγ production and the avoidance of anergy [27,28]. Responding to stimulation under favorable conditions, D cyclins are expressed sequentially starting in late G_0_/early G_1 _during the normal progression of the cell cycle [28,29]. Next, Cyclin E emerges during late G_1 _phase following degradation/sequestration of the CDKIs p27^Kip1 ^and p21^Cip1^. The CDKIs p27^Kip1 ^and p21^Cip1 ^are instrumental in a coordinated G_1 _to S phase transition "holding" the cellular machinery in place until the cyclins and CDKs are at the proper levels and activation state. Cyclins partner with their cyclin dependent kinase to sequentially phosphorylate Rb during G_1 _progression. Hyperphosphorylation of Rb and release of E2F transcription factors signals the irreversible commitment to S phase and cell cycle progression [28,29].

There are at least two broad categories of CD4^+^CD25^+ ^Treg cells, natural Treg cells and adaptive (or induced) Tregs [30,31]. Natural Treg cells originate in the thymus and reside in peripheral lymph tissues to prevent autoimmune responses [32,33]. Adaptive Treg cells are phenotypically indistinguishable from natural Treg cells and modulate immune responses to microbial pathogens including bacteria, viruses, fungi, and intracellular parasites [34-36]. A third population of regulatory cells, Foxp3^+^CD8^+ ^regulatory lymphocytes has also been described [37-40]. The derivation of Foxp3^+^CD8^+ ^regulatory lymphocytes is not completely understood, however like their CD4^+^Foxp3^+ ^counterparts, it is plausible that there is both a "natural" and "adaptive" subset of these cells. Foxp3 is a forkhead transcription factor which binds DNA adjacent to NFAT sites and is essential to the development of CD4^+^CD25^+ ^regulatory T cells [41-43]. We and others have shown that Foxp3 expression can be induced in CD4^+^CD25^- ^target cells under certain conditions and that these induced Foxp3^+ ^cells exhibit suppressor activity [44,45]. Stable Foxp3 expression is essential for Treg development and function, but is not exclusive to regulatory T cells, as transient or unstable Foxp3 expression has been observed in other T cell subsets, suggesting that Foxp3 may play other roles in T cell homeostasis [46-48].

Because activated Treg cells are known to induce anergy in T cell targets and because FIV infection activates Treg cells, we asked whether activated Treg cells from FIV^+ ^cats altered the expression of cyclins, cyclin-dependent kinases and cyclin-dependent kinase inhibitors that regulate anergy in CD8^+ ^target cells. In FIV infection, CD8^+ ^lymphocytes display an activated phenotype, yet have compromised effector function, reminiscent of anergy [3,13,14]. It is likely that CD8^+ ^lymphocytes receive both stimulatory and inhibitory signals, leading to a complex convergence of intracellular signaling events. We therefore systematically evaluated cell cycle proteins, starting with G_1 _phase proteins, in an effort to determine when and if anergy occurs in CD8^+ ^lymphocyte targets following their interaction with activated Treg cells. To further define the relationship between activated Treg cells and CD8^+ ^targets in FIV infected cats, we asked if Treg cells from chronically infected FIV^+ ^cats might also induce suppressor function in CD8^+ ^target cells following co-culture.

## Results

### Cyclin D_3 _production is decreased and cyclin E production is increased in CD8^+ ^targets from FIV^+ ^cats following CD4^+^CD25^+ ^co-culture

**Figure 2:**
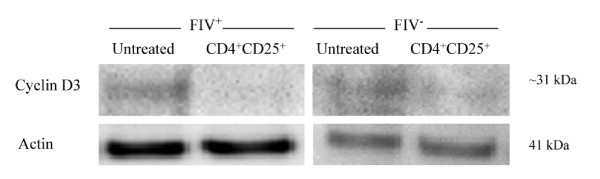
**CD8^+ ^lymphocyte Cyclin D_3 _production in FIV^+ ^and FIV^- ^cats following CD4^+^CD25^+ ^co-culture**. Cyclin D_3 _typically assembles with CDK4 or CDK6 during mid G_1 _phase and reaches maximal production during late G_1_/early S phase. CD8^+ ^LN cells from either FIV^+ ^(left) or FIV^- ^(right) cats were either untreated (first column), or co-cultured with autologous CD4^+^CD25^+ ^Treg cells (second column). Shown above is a representative blot for experiments from FIV^+ ^(n = 4) and FIV^- ^(n = 2) cats. In both FIV^+ ^and FIV^- ^control cats, the mean cyclin D3 production was reduced following a twelve hour incubation with CD4^+^CD25^+ ^Treg cells.

To examine the effect of FIV infection on cell cycle regulatory proteins that could explain T cell-mediated anergy, cyclin D_3 _was examined first during sequential evaluation of cell cycle proteins in CD8^+ ^target cells. In T lymphocytes, cyclin D_3 _typically assembles with CDK4 or CDK6 during mid G_1 _phase and reaches maximal production during late G_1_/early S phase (Figure 1[28,29]). Lymph node CD8^+ ^cells from either FIV^+ ^or FIV^- ^cats were untreated or co-cultured with CD4^+^CD25^+ ^Treg cells. In both FIV^+ ^and FIV^- ^cats, cyclin D_3 _was modestly reduced in CD8^+ ^cells following a twelve hour co-culture with CD4^+^CD25^+ ^Treg cells (Figure 2, Additional file 1, Table S1).

**Figure 3:**
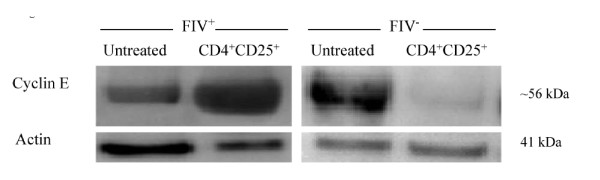
**CD8^+ ^lymphocyte Cyclin E production in FIV^+ ^and FIV^- ^cats following CD4^+^CD25^+ ^co-culture**. Cyclin E production begins during late G_1 _phase and peaks during early to mid S phase. CD8^+ ^LN cells from either FIV^+ ^(left) or FIV^- ^(right) cats were either untreated (first column) or co-cultured with autologous CD4^+^CD25^+ ^Treg cells (second column). Shown above is a representative blot for experiments from FIV^+ ^(n = 4) and FIV^- ^(n = 2) cats. The mean cyclin E production was increased greater than two-fold in FIV^+ ^cats following a twelve hour CD4^+^CD25^+ ^co-culture and decreased approximately one-fold in FIV^- ^cats.

Because cyclin E emerges in late G_1 _to facilitate G_1 _to S transition, we asked whether there was any change in cyclin E in CD8^+ ^targets following CD4^+^CD25^+ ^co-culture. As shown in Figure 3 and supplemental table 1, there was a greater than 2 fold increase in cyclin E production in CD8^+ ^targets from FIV^+ ^cats with a moderate decrease in cyclin E production in FIV^- ^control cats.

### The CDKI p21^Cip1 ^is increased in CD8^+ ^target cells from chronically infected FIV^+ ^cats following CD4^+^CD25^+ ^co-culture

**Figure 4:**
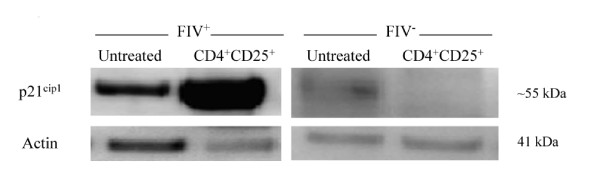
**CD8^+ ^lymphocyte p21^cip1 ^production in FIV^+ ^and FIV^- ^cats following CD4^+^CD25^+ ^co-culture**. Levels of the CDKI p21^cip1 ^begin to increase during G_0 _phase and reach maximal production in late G_1_/early S phase. However, p21^cip1 ^is also increased in anergic T cells; thereby preventing the G to S phase transition. CD8^+ ^LN cells from either FIV^+ ^(left) or FIV^- ^(right) cats were either untreated (first column) or co-cultured with autologous CD4^+^CD25^+ ^Treg cells (second column). Shown above is a representative blot for experiments from FIV^+ ^(n = 4) and FIV^- ^(n = 2) cats. p21^cip1 ^production was increased by approximately 1.7 fold in FIV^+ ^cats following a twelve hour CD4^+^CD25^+ ^co-culture.

We asked if activated Treg cells from FIV-infected cats might induce CDKI production in lymphocyte targets, because increased CDKI production correlates with cell cycle arrest in lymphocytes [28,29,49,50]. The Ink 4 family of CDKIs, such as p15^Ink4b^, can antagonize the assembly of cyclin D-dependent kinases [29]. The Cip/Kip family of CDKIs includes p21^Cip1 ^and p27^Kip1 ^which bind cyclins D, E, and A [29]. The CDKI p21^Cip1 ^helps control the activation and survival of autoreactive T cells and overproduction is associated with G_1 _cell cycle arrest [28,50,51]. There was greater than a 1.5 fold increase in p21^Cip1 ^in CD8^+ ^targets from FIV^+ ^cats following a twelve-hour CD4^+^CD25^+ ^co-culture, while only a slight reduction in p21^Cip1 ^was observed in CD8^+ ^targets from FIV^- ^cats following a twelve-hour CD4^+^CD25^+ ^co-culture (Figure 4, Additional file 1, Table S1). The levels of both p15^Ink4b ^and p27^Kip1 ^production in CD8^+ ^targets following CD4^+^CD25^+ ^co-culture were unchanged (Additional file 2, Figure S1).

### Hyperphosphorylation of Rb is not evident in CD8^+ ^target cells following CD4^+^CD25^+ ^co-culture

**Figure 5:**
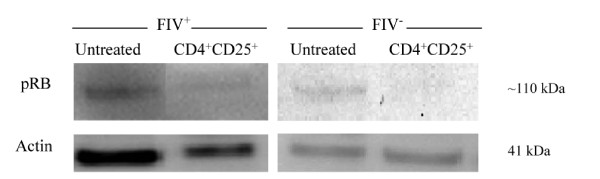
**CD8^+ ^lymphocyte Rb phosphorylation in FIV^+ ^and FIV^- ^cats following CD4^+^CD25^+ ^co-culture**. Hyperphosphorylation of Rb by cyclin/CDK complexes and subsequent separation of E2F proteins from Rb signals the irreversible commitment of the cell to S phase; while lack of Rb phosphorylation suggest either quiescence (G_0_) or anergy (G_1 _cell cycle arrest). As depicted here, CD8^+ ^LN cells from either FIV^+ ^(left) or FIV^- ^(right) cats were either untreated (first column), or co-cultured with autologous CD4^+^CD25^+ ^Treg cells (second column). Shown above is a representative blot for experiments from FIV^+ ^(n = 4) and FIV^- ^(n = 2) cats. There was a lack of Rb phosphorylation in both FIV^+ ^and FIV^- ^cats following an eighteen hour CD4^+^CD25^+ ^co-culture.

**Figure 6:**
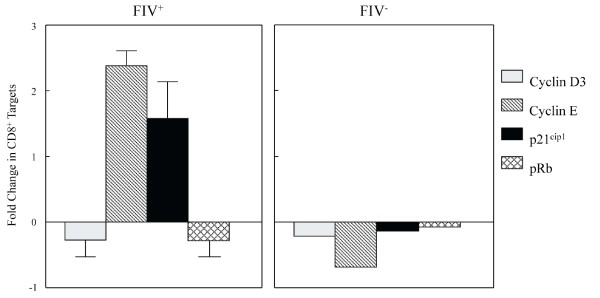
**A summary of the relative production levels of Cyclins D and E, the CDKI p21^cip1^, and Rb in CD8^+ ^lymphocytes from FIV^+ ^and FIV^- ^cats following CD4^+^CD25^+ ^co-culture**. FIV^+ ^cats exhibit a decrease in cyclin D_3 _with increases in both cyclin E and p21^cip1^. This pattern is consistent with a cell that is in either late G_1 _or early S phase of the cell cycle (as shown in Figure 1). The lack of Rb phosphorylation suggests that the CD8^+ ^lymphocytes from FIV^+ ^cats are in late G_1 _cell cycle arrest following co-culture with activated CD4^+^CD25^+ ^lymphocytes. For FIV^+ ^cats, each bar represents the mean (+ SEM) of four separate experiments, for FIV^- ^cats each bar represents the mean of two separate experiments.

Collectively, the results of Figures 2, 3 and 4 demonstrate that cyclin D_3 _levels have declined while cyclin E and p21^Cip1 ^levels have increased. This profile could be consistent with one of two outcomes: either the target cell has progressed to S phase or the cell has undergone late G_1 _cell cycle arrest. In an effort to clearly delineate late G_1 _cell cycle arrest from early S phase transition, we examined Rb phosphorylation status. Hyperphosphorylation of Rb allows release of the E2F family of transcription factors and signals irreversible S phase commitment [27,29]. Rb protein hyperphosphorylation was not evident in CD8^+ ^target cells following an eighteen hour CD4^+^CD25^+ ^co-culture (Figure 5, Additional file 1, Table S1). In sum, the findings of Figures 2, 3, 4 and 5 are most consistent with CD4^+^CD25^+ ^Treg-induced anergy in CD8^+ ^target cells from FIV^+ ^cats (Figure 6).

### IL-2 mRNA expression is reduced in CD8^+ ^target cells from chronically infected FIV^+ ^cats following CD4^+^CD25^+ ^co-culture

**Figure 7:**
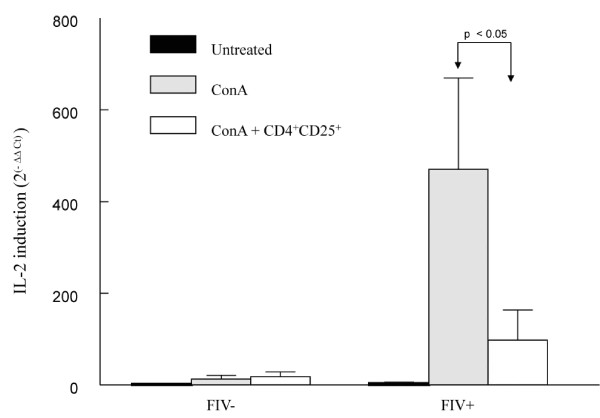
**IL-2 mRNA is decreased in CD8^+ ^lymphocyte targets following CD4^+^CD25^+ ^co-culture**. CD8^+ ^lymphocytes from FIV^- ^or FIV^+ ^cats were either untreated, ConA stimulated (5 ug/ml), or CD8^+ ^targets were ConA stimulated for two hours prior to autologous CD4^+^CD25^+ ^Treg co-culture. After twenty-four hours, RNA was isolated and reverse transcription RT PCR was performed on all sample groups. For the CD8^+^/CD4^+^CD25^+ ^co-culture, CD4^+^CD25^+ ^cells were depleted by FACS prior to RNA isolation. IL-2 mRNA was decreased by approximately four-fold in ConA stimulated, CD8^+ ^lymphocytes from FIV^+ ^cats following CD4^+^CD25^+ ^co-culture (p < 0.05, arrows). Each bar represents the mean + SEM for six experiments.

Lymphocyte activation is regulated by cyclin-dependent kinases that stimulate the production of IL-2 mRNA [27,28,50,52,53]. Autocrine and paracrine production of IL-2 is critical to lymphocyte expansion, differentiation, and the avoidance of anergy [54-57]. Therefore, we examined IL-2 mRNA to validate the findings in Figures 2, 3, 4, 5 and 6. There was a greater than four-fold reduction in IL-2 mRNA in stimulated CD8^+ ^lymphocytes from FIV^+ ^cats following CD4^+^CD25^+ ^co-culture as compared to stimulated CD8^+ ^lymphocytes alone, consistent with the induction of anergy (Figure 7).

### Foxp3 expression is increased in CD8^+ ^targets from FIV^+ ^cats following CD4^+^CD25^+ ^co-culture, but CD8^+ ^target cells lack suppressor function

**Figure 8:**
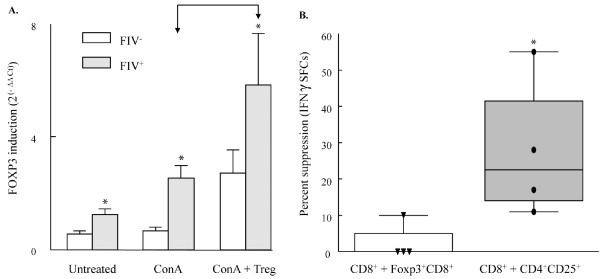
**CD4^+^CD25^+ ^Treg cells induce Foxp3 expression but not suppressor function in CD8^+ ^lymphocyte targets**. (A). CD8^+ ^lymphocytes from FIV^- ^or FIV^+ ^cats were either untreated, ConA stimulated (5 ug/ml) or ConA stimulated for two hours then co-cultured with autologous CD4^+^CD25^+ ^Treg cells for twenty-four hours. After twenty-four hours, RNA was isolated and reverse transcription RT PCR was performed on all sample groups. For the CD8^+^/CD4^+^CD25^+ ^co-cultures, CD4^+^CD25^+ ^cells were depleted by FACS prior to RNA isolation. Foxp3 induction was significantly higher in all treatment groups from FIV^+ ^cats when compared to FIV^- ^cats (asterisks, p < 0.05) and in ConA stimulated, CD8^+ ^lymphocytes following CD4^+^CD25^+ ^co- culture when compared to ConA stimulation alone (p < 0.05 arrows). Each bar represents the mean + SEM for six experiments. (B). CD8^+ ^lymphocytes from FIV^+ ^cats were ConA stimulated then co-cultured with autologous CD4^+^CD25^+ ^cells to induce Foxp3 expression as described in part A. Following co-culture, CD4^+^CD25^+ ^cells and CD8^+ ^target cells were re-sorted and then co-cultured with ConA stimulated (2 hours before co-culture), autologous CD8^+ ^lymphocytes for forty-eight hours in IFNγ ELISpot plates. Percent suppression was calculated by the following: ConA stimulated CD8^+ ^lymphocyte SFCs ÷ ConA stimulated CD8^+ ^lymphocytes + Foxp3^+ ^CD8^+ ^lymphocytes SFCs or ConA stimulated CD8^+ ^lymphocyte SFCs ÷ ConA stimulated CD8^+ ^lymphocytes + CD4^+^CD25^+ ^lymphocytes SFCs. The box-whisker plots represent 5th and 95th percentiles (whisker), 25th and 75th percentiles (box) and median of percent suppression, dots represent individual cats. There was little suppression evident when CD8^+ ^targets were co-cultured with Foxp3^+^CD8^+ ^cells. As expected, CD4^+^CD25^+ ^lymphocytes suppressed IFNγ production in CD8^+ ^targets (p < 0.01, asterisks).

We asked whether the CD8^+ ^target cells from FIV^+ ^cats shown in Figures 2, 3, 4, 5, 6 and 7 might upregulate Foxp3 and exhibit suppression of autologous CD8^+ ^responses. As shown in Figure 8a, Foxp3 induction in FIV^+ ^cats was maximal in ConA stimulated (5 ug/ml), CD8^+ ^lymphocytes following a 24 hour CD4^+^CD25^+ ^co-culture (p < 0.05). Foxp3 levels did not increase any further following a 48 hour co-culture (data not shown). To assess suppressive potential following co-culture, CD8^+ ^target cells and CD4^+^CD25^+ ^Treg cells were then re-sorted and combined with autologous CD8^+ ^lymphocytes to assay IFNγ production. Figure 8b demonstrates that CD4^+^CD25^+ ^cells from FIV^+ ^cats inhibited CD8^+ ^IFNγ spot forming cells (SFCs) by approximately twenty-five percent. However, in the same experiment, CD8^+ ^lymphocytes previously co-cultured with the same CD4^+^CD25^+ ^cells lacked suppressor function despite upregulation of Foxp3.

## Discussion

The mechanisms underlying T cell immune dysfunction during the course of AIDS lentiviral infections are still not completely understood. One of the more puzzling aspects of these infections is the presence of lymphocytes that appear to be activated yet exhibit compromised effector function [14,58]. This laboratory and others have documented Treg mediated immune suppression of both CD4^+^CD25^- ^and CD8^+ ^lymphocytes during acute and chronic AIDS lentiviral infection [1-3,7,8]. Based upon these data, the authors have explored the intracellular events in the CD8^+ ^target cells, following co-culture with CD4^+^CD25^+ ^Treg cells, for a clearer understanding of what may contribute to CD8^+ ^immune dysfunction. As CD8^+ ^lymphocytes are important for both the elimination of acute viral infections and control of chronic viral infections, understanding Treg-mediated CD8^+ ^anergy may be one of the keys to understanding AIDS associated immune dysfunction.

As T cell anergy appears to be an important component to virus induced immune dysfunction, we studied production of molecules that regulate both cell cycle progression and cellular anergy. Because the control of cell cycle progression versus cell cycle anergy is regulated by the relative production of selected cell cycle proteins during the G_1 _to S phase transition; we examined a number of these proteins in CD8^+ ^T cells anergized by contact with activated CD4^+^CD25^+ ^Treg cells from FIV infected cats. As shown in Figure 2, there was a modest decrease in cyclin D_3 _following a twelve hour Treg co-culture. In general, cyclin D_3 _levels are expected to increase during the progression from G_1 _to S phase, suggesting that the CD8^+ ^target cells had either progressed well into S phase, or had begun G_1 _cell cycle arrest [28]. Cyclin E emerges during the progression from G_1 _to S phase and Figure 3 clearly shows an increase in cyclin E in FIV^+ ^cats following a twelve hour Treg co-culture, while there was a moderate decrease in cyclin E in FIV^- ^cats. Cyclin A emerges during early S phase and progressively increases during S phase [28]. There was no change in cyclin A activity evident following an eighteen hour Treg co-culture. The lack of increased cyclin A activity suggests that the cells were in very late G_1 _cell cycle arrest (Additional file 2, Figure S1). Next, the CDKI p21^cip1 ^was examined. This CDKI is reported to have a complex role in cell cycle regulation by facilitating the activity of the D cyclin family, while inhibiting the activity of cyclin E [28,49]. As shown in Figure 4 and Figure 6, in CD8^+ ^target cells from FIV^+ ^cats, p21^cip1 ^was increased by approximately 1.7 fold, following co-culture with CD4^+^CD25^+ ^Treg cells. During the course of G_1 _progression, Rb is sequentially phosphorylated at different sites by cyclin/CDK complexes, which facilitates the release of E2F transcription factors, marking the irreversible commitment to S phase [29]. Therefore, increases in intracellular cyclin E, should be followed by Rb hyperphosphorylation if the cell progresses into S phase. As shown in Figure 5, there was no Rb hyper-phosphorylation evident following Treg co-culture, suggesting that both cyclin D and cyclin E failed to phosphorylate Rb.

In fibroblasts and CD4^+ ^lymphocytes during normal cell cycle progression, p21^cip1 ^reaches maximal production levels during S phase [28,59]. However, in different models of liver disease, increased p21^cip1 ^production is associated with G_1 _cell cycle arrest [60]. Conversely, p21^cip1 ^knockout mice exhibit shorter G_1 _to S phase transition times and greater proliferative capacity [49]. A recent report by Bergamashi et al [61] has demonstrated increased p21^cip1 ^production in macrophages from HIV-infected individuals that may be associated with inhibition of viral replication within the macrophage. These findings suggest that increased p21^cip1 ^production in CD8^+ ^targets is likely associated with late G_1 _cell cycle arrest. The upregulation of p21^cip1 ^may provide a beneficial effect to the host by creating a poor environment for viral replication while conversely contributing to the development of immunodeficiency by halting CD8^+ ^effector and proliferative responses.

The findings in Figures 2, 3, 4, 5 and 6 are consistent with late G_1 _cell cycle arrest and anergy. To further characterize this interaction, we asked if Treg cells from FIV^+ ^cats would suppress IL-2 mRNA expression in autologous CD8^+ ^targets. The ability to produce IL-2 is a reflection of lymphocyte activation, because it requires a convergence of intracellular events, including cyclin-dependent kinase activation of E2F transcription factors [27,28,50,52,53]. Initially, exogenous signals are critical to stimulating the CD8^+ ^cell to produce IL-2 for lymphocyte expansion, differentiation, and the avoidance of anergy [54-57]. As shown in Figure 7, CD8^+ ^lymphocytes were stimulated with ConA to promote IL-2 production. Lymphocytes from FIV^- ^cats exhibited very modest increases in IL-2 mRNA following ConA stimulation, likely because these cats were SPF animals with little antigenic exposure and a relatively quiescent immune system. This is similar to our previous observation that CD8^+ ^lymphocytes from FIV^-^, SPF cats produce very little IFNγ mRNA following ConA stimulation [3]. The CD8^+ ^lymphocytes from FIV^+ ^cats exhibited a marked increase in IL-2 mRNA following ConA stimulation which was then markedly decreased following co-culture with CD4^+^CD25^+ ^Treg cells. Taken together, the findings of decreased cyclin D_3 _production, increased cyclin E and p21^cip1 ^production, lack of cyclin A production, lack of Rb phosphorylation, combined with suppression of IL-2 mRNA in CD8^+ ^targets suggests that Treg cells from FIV^+ ^cats are able to induce very late G_1 _cell cycle arrest in CD8^+ ^targets. This also may help to explain, in part, why CD8^+ ^lymphocytes from FIV^+ ^cats display an activated phenotype yet have marginal effector function.

There is a degree of plasticity in T helper versus Treg phenotype and function; for example, under appropriate stimulating conditions, CD4^+ ^T cells exhibiting T helper phenotype and function can be converted into Treg (or Treg "like") cells [44,45]. As demonstrated in murine models and in FIV infection, these converted cells express Foxp3 and suppress T helper effector responses [44,45]. There is also evidence for expansion of CD8^+^Foxp3^+ ^suppressor cells in the SIV lentivirus model [40]. Therefore, we asked if Foxp3 might also be up-regulated in CD8^+ ^targets from FIV^+ ^cats following Treg co-culture. We observed CD8^+ ^target cell up-regulation of Foxp3 following CD4^+^CD25^+ ^co-culture, however, these target cells lacked suppressor function (Figure 8). Our results are consistent with those also reported by Dieckmann et al. [62] who demonstrated that activated Treg cells co-cultured with CD8^+ ^target cells suppressed effector function and induced anergy in CD8^+ ^targets, but did not convert these cells into CD8^+ ^suppressor cells. Recent reports demonstrate that Foxp3 expression can be transiently induced in human CD4^+ ^and CD8^+ ^T lymphocyte targets without these cells exhibiting regulatory function; however, the function of Foxp3 in these target cells in unclear [46-48]. Further investigation is needed to clarify the role of Foxp3 expression in these cells.

## Conclusions

Analysis of proteins involved in cell cycle regulation is consistent with late G_1 _cell cycle arrest in CD8^+ ^targets from FIV^+ ^cats following CD4^+^CD25^+^/CD8^+ ^co-culture (Figures 2, 3, 4, 5 and 6). Figure 7 clearly shows Treg-mediated suppression of IL-2 mRNA production in CD8^+ ^targets and we have recently reported reduced IFNγ production in CD8^+ ^target cells from FIV^+ ^cats following CD4^+^CD25^+ ^Treg co-culture [3]. Collectively, these data suggest Treg-mediated inhibition of both effector and proliferative functions in CD8^+ ^targets from FIV^+ ^cats. Previous work suggests that CD4^+^CD25^+ ^Treg cells are activated early and progressively during the course of FIV infection and that inhibition of CD4^+^CD25^- ^and CD8^+ ^effector responses occurs early and progressively during the course of FIV infection [1-3]. Further understanding of how Treg cells inhibit CD8^+ ^antiviral function and CD4^+ ^T helper function during the course of FIV infection will help to clarify how lentiviruses establish and maintain a persistent infection and may offer insight into the development of novel vaccination and treatment strategies.

## Methods

### Cats

Specific pathogen free (SPF) cats were obtained from Liberty Research, Inc. (Waverly, NY) and housed in the Laboratory Animal Resource Facility at the College of Veterinary Medicine, North Carolina State University. FIV infected cats were housed separately from uninfected control cats. Protocols were approved by the North Carolina State University Institutional Animal Care and Use Committee.

### Infection with FIV

The NCSU_1 _isolate of FIV was originally obtained from a naturally infected cat at the North Carolina State University College of Veterinary Medicine and has been described in detail elsewhere [63]. Virus inoculum was grown as a single tissue culture passage in an IL2-dependent feline CD4^+ ^cell line (FCD4-Ecells) as previously described [64]. The cats were infected intravenously with 1 × 10^5 ^TCID_50 _of cell-free virus culture and FIV infection was confirmed on serum samples by using a commercially available ELISA Kit (IDEXX Laboratories). The cats had been infected for approximately 2 years prior to these experiments. Plasma viremia was not assessed at the time of lymphocyte collection for the experiments outlined in Figures 2, 3, 4, 5, 6, 7 and 8. The FIV^+ ^cats in this study had normal lymphocyte counts (mean = 2812/μl) with an inverted CD4:CD8 ratio (mean = 0.61). Control cats were age matched uninfected SPF cats.

### Sample collection

Lymphocytes were harvested either by LN excision or following euthanasia. Lymph node biopsies were performed as previously described [2,65]. Following collection, lymph nodes were processed into a single cell suspension for purification of lymphocyte subsets.

### Antibodies

Murine monoclonal anti-feline CD4 (mAb 30A), CD8 (mAb 3.357) and CD25 (mAb 9F23) were produced in our laboratory [66]. The anti-feline CD25 (mAb 9F23) was originally provided by K. Ohno (University of Tokyo). The antibodies were conjugated to FITC (anti-CD8, anti-CD25), PE (anti-CD4, anti-CD8) or biotin (anti-CD8) (developed with Streptavidin/PerCP).

### Lymphocyte sorting and culture

Lymphocytes were sorted into CD8^+ ^and CD4^+^CD25^+ ^populations by FACS, using a Moflo high speed cell sorter. Populations were ~99% pure. Lymphocyte cultures were maintained in serum restricted media (1.0% FBS) in 12 well, flat bottom plates. Following CD8^+^/CD4^+^CD25^+ ^co-cultures, CD8^+ ^and CD4^+^CD25^+ ^cells were then re-sorted by FACS and examined by western blot, PCR or ELISpot.

### Reverse transcription real time PCR

2 × 10^6 ^CD8^+ ^lymphocytes from FIV^- ^and FIV^+ ^cats were untreated, ConA stimulated (5 ug/ml) for two hours and washed, or ConA stimulated for two hours and washed followed by co-culture with CD4^+^CD25^+ ^cells for 24 hrs (CD4^+^CD25^+ ^to CD8^+ ^ratio = 1:1). Following CD8^+^/CD4^+^CD25^+ ^co-cultures, CD8^+ ^and CD4^+^CD25^+ ^cells were then re-sorted by FACS. RNA from cell cultures was isolated using the Qiagen RNeasy plus Mini Kit and reverse transcription was performed using the Promega Reverse Transcription System, following the manufacturer's instructions for both. This reaction was followed by a real-time PCR step using the universal Taqman PCR Mastermix (Applied Biosystems) and the Qiagen Quantitect Sybr Green PCR Kit (probe). The reactions were run in duplicates in 96 well plates. The fold induction was calculated by using the ΔΔCt value, where Fold Induction = 2 - (ΔΔCt), as described by Winer et al [67]. PBMCs from an FIV negative cat and GAPDH as the internal control were used as the calibration sample value in the ΔΔCt equation. The feline specific IL-2, Foxp3, and GAPDH primer sequences utilized for the real time PCR reaction were as follows: IL-2 (forward ACA GTG CAC CTG CTT CAA GCT CT-3' and reverse CCT GGA GAG TTT GGG GTT CTC AGG), Foxp3 (forward GCC TGC CAC CTG GAA TCA AC and reverse GTG TGC TGG GGC TTG GGA), and GAPDH (forward GGA GAA GGC TGG GGC TCA C and reverse GGT GCA GGA GGC ATT GCT GA).

### Western Blotting

Approximately 4 × 10^6 ^CD8^+ ^FACS purified CD8^+ ^and CD4^+^CD25^+ ^lymphocytes were harvested for each treatment group. For co-culture experiments, CD8^+ ^and CD4^+^CD25^+ ^were co-cultured at a 1:1 ratio and then re-sorted using FACS (~99% purity). The CD8^+ ^cells were then lysed with NP-40 and separated by SDS-Page. The blots were analyzed using anti-cyclin D3 (Cell Signaling Technologies #2936), anti-Cyclin E (Cell Signaling Technologies #4129), anti-p21 (Novus Biologicals #NB-120-14061), and anti-Rb (Cell Signaling Technologies #9308), followed by HRP-conjugated goat anti-mouse IgG1 and detected by chemiluminescence. The blots were then stripped and re-probed with anti-actin and HRP-conjugated goat anti-mouse. For each treatment group, actin and the protein in question were evaluated by photodensitometry and normalized using the VersaDoc imaging system (Bio-Rad Laboratories). For reporting of fold change, each treatment group was compared to unstimulated CD8^+ ^controls which were assigned a value of 1.

### IFNγ ELISpot

Following co-culture, CD4^+^CD25^+ ^cells and CD8^+ ^target cells were re-sorted, assessed by trypan blue staining for viability (<10% positive), and then cultured alone (2.5 × 10^5 ^per well) or co-cultured with ConA stimulated autologous CD8^+ ^lymphocytes (1:1 ratio) for 48 hrs in pre-coated 96 well ELISpot plates (monoclonal anti-feline IFNγ, R and D systems). The ConA stimulated CD8^+ ^lymphocyte targets were stimulated for two hours then washed prior to co-culture. The plates were incubated for 24 hours, stained with detection antibody, and developed per the manufacturer's instructions. Once dry, each well was counted with an automated ELISpot reader for quantification of spot forming cells (SFC) per number of cells plated in each well. Percent suppression was calculated by the following: (1) ConA stimulated CD8^+ ^lymphocyte SFCs ÷ ConA stimulated CD8^+ ^lymphocytes + Foxp3^+ ^CD8^+ ^lymphocytes SFCs or (2) ConA stimulated CD8^+ ^lymphocyte SFCs ÷ ConA stimulated CD8^+ ^lymphocytes + CD4^+^CD25^+ ^lymphocytes SFCs. CD4^+^CD25^+ ^lymphocytes alone and CD8^+^Foxp3^+ ^alone did not produce any IFNγ SFCs.

## Competing interests

The authors declare that they have no competing interests.

## Authors' contributions

JF carried out all of the studies contained in this manuscript and drafted the manuscript. WT assisted with study design, data interpretation and manuscript revisions. MT assisted with study design, data interpretation and manuscript revisions. All authors read and approved the final manuscript.

## Supplementary Material

Additional File 1 **Table S1: Fold change in the production of cyclins D and E, the CDKI p21^cip1^, and Rb in CD8^+ ^lymphocytes from FIV^+ ^and FIV^- ^cats following CD4^+^CD25^+ ^co-culture**. The values (rows) for individual FIV^+ ^(n = 4) and FIV^- ^(n = 2) cats are shown for each protein (columns). The last value for each group is the mean fold change. As reported in the methods, the fold change in CD8^+ ^target cells was calculated by comparing the protein in question following co-culture to CD8^+ ^target cells alone. Click here for file

Additional File 2 **Figure S1: Cyclin A, p15^Ink4b ^and p27^Kip1 ^protein production in CD8^+ ^lymphocytes following CD4^+^CD25^+ ^co-culture**. The levels of these three proteins remained unchanged in CD8^+ ^targets following CD4^+^CD25^+ ^co-culture. The results are representative of two (cyclin A) or four (p15^Ink4b ^and p27^Kip1^) separate experiments from FIV^+ ^cats. Click here for file
