# Supplementary figures and images for: CD4+CD25+ T regulatory cells from FIV+ cats induce a unique anergic profile in CD8+ lymphocyte targets
Source: Retrovirology. 2010 Nov 19;7:97. doi: 10.1186/1742-4690-7-97 (PMC2997086; doi:10.1186/1742-4690-7-97)

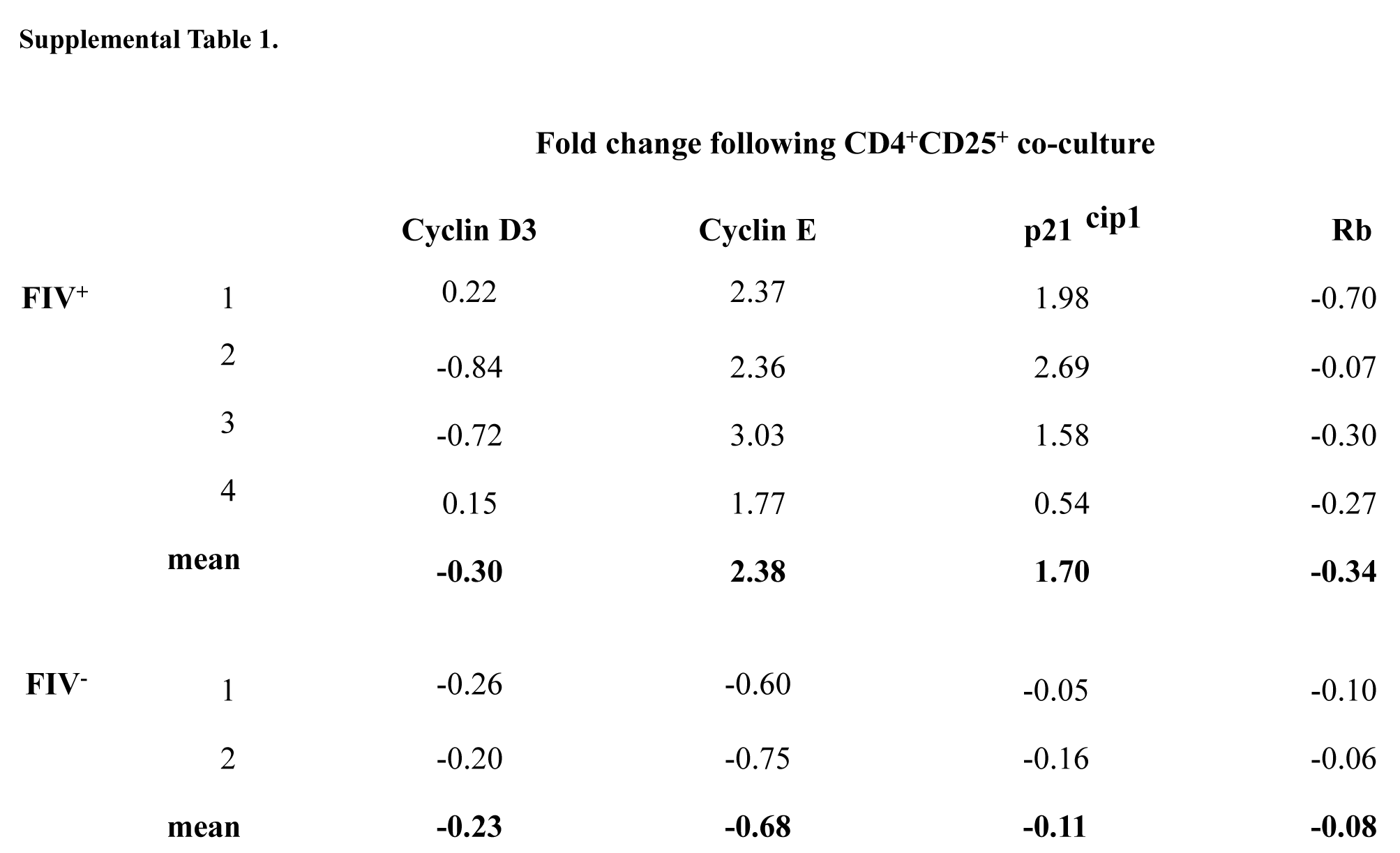

Supplement: Additional File 1 — Table S1: Fold change in the production of cyclins D and E, the CDKI p21cip1, and Rb in CD8+ lymphocytes from FIV+ and FIV- cats following CD4+CD25+ co-culture. The values (rows) for individual FIV+ (n = 4) and FIV- (n = 2) cats are shown for each protein (columns). The last value for each group is the mean fold change. As reported in the methods, the fold change in CD8+ target cells was calculated by comparing the protein in question following co-culture to CD8+ target cells alone. [file 1742-4690-7-97-S1.PNG]

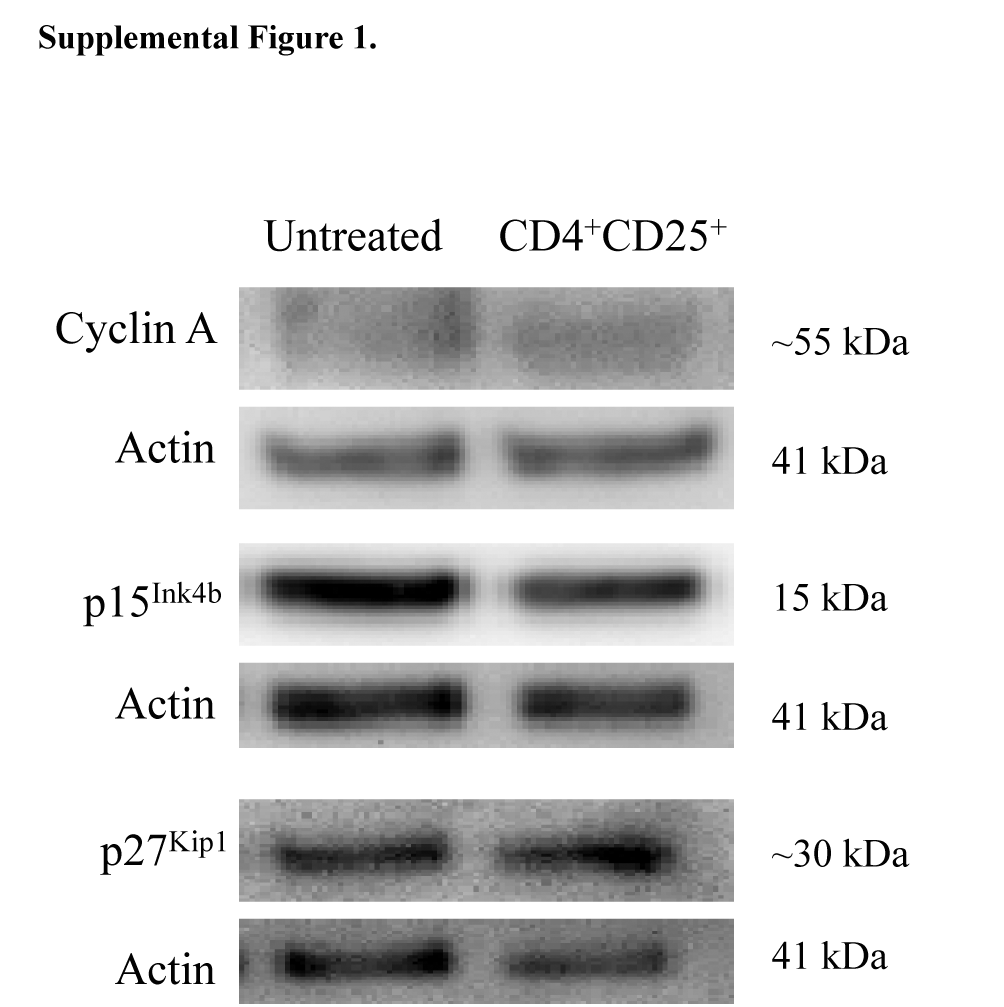

Supplement: Additional File 2 — Figure S1: Cyclin A, p15Ink4b and p27Kip1 protein production in CD8+ lymphocytes following CD4+CD25+ co-culture. The levels of these three proteins remained unchanged in CD8+ targets following CD4+CD25+ co-culture. The results are representative of two (cyclin A) or four (p15Ink4b and p27Kip1) separate experiments from FIV+ cats. [file 1742-4690-7-97-S2.PNG]
